# Intersubjectivity and interaction as crucial for understanding the moral role of shame: a critique of TOSCA-based shame research

**DOI:** 10.3389/fpsyg.2014.00814

**Published:** 2014-07-29

**Authors:** Alba Montes Sánchez

**Affiliations:** ^1^Center for Subjectivity Research, Department of Media, Cognition and Communication, University of CopenhagenCopenhagen, Denmark; ^2^Departamento de Humanidades: Filosofía, Lenguaje y Literatura, Universidad Carlos III de MadridMadrid, Spain

**Keywords:** shame, guilt, moral emotions, relationality, intersubjectivity

In recent years, a view on two key moral emotions, shame and guilt, seems to be establishing itself in some sectors of psychology, based mainly on the research of Tangney and Dearing (2004) and their “Test of Self-Conscious Affect” (TOSCA). On this view, guilt is a productive force in our moral lives, while shame is morally counterproductive and psychologically harmful. Therefore, one should cultivate guilt and fight shame. But this conclusion is problematic for two main reasons, among others. On the one hand, the distinction that grounds it is too simplistic: the boundary between guilt and shame is far more blurry and complex than this account acknowledges. On the other hand, it operates on a functionalistic definition of morality, where “moral” means “prosocial,” which is ultimately insufficient to account for the moral role of these emotions. The functionalistic approach neither does justice to the self-conscious aspects of guilt and shame nor to the interactive dimensions of morality, as a shared practice we engage in with others (Calhoun, 2004).

## Tangney and dearing's account

According to Tangney and Dearing (2004), the main difference between shame and guilt lies in their objects of focus: shame focuses on the ashamed self, while guilt focuses on behavior. In shame we feel bad about the way we are, about some characteristic or feature of ours, while in guilt we feel bad about our actions or omissions, about having done something wrong, broken a norm or harmed somebody. On this view, because self is perceived as much more difficult to change or undo than behavior, shame leads to antisocial tendencies (shunning contact with others, lashing out in anger), and ultimately to low self-esteem, depression and addictions. In contrast, guilt motivates prosocial efforts (apologizing, attempting to undo or compensate the harm done), and it is not correlated to low self-esteem or addictions. Therefore guilt is seen as productive and shame as counterproductive. However, another finding of Tangney's should give us pause. In a study of incarcerated offenders, Tangney and Stuewig claim that the only people who have no capacity for shame are psychopaths; therefore they conclude that in “extreme populations” some shame is better than the absence of any self-evaluative emotion, as it offers a ray of hope for social reintegration (Tangney and Stuewig, 2004, p. 327). But if shame is thus in some way connected to moral sensibility, why should this conclusion only hold for “extreme populations”?

## Problems with the distinction between shame and guilt

Let us take a closer look at the problems entailed by this account. First, although Tangney and Dearing's definitions of shame and guilt, based on the work of Helen Block Lewis (1971), are widely accepted and indicate a helpful distinction, they should be handled with care. Tangney et al. (1996) have shown that people tend to have trouble distinguishing between shame and guilt (while they find it much easier to distinguish between shame and embarrassment). Dearing and Tangney (2011, pp. 9–11) explain this as an error of judgment or a confusion on the part of therapists or clients, but I disagree. Dearing and Tangney present these emotions as two perfectly discrete processes that produce very different responses and have very different functions, but this is very dubiously the case. Guilt and shame are complex self-conscious emotions, with a high degree of cognitive specification and wide variations from culture to culture. They are in the same emotional territory, they share a vast phenomenal ground and work together in many ways. Some authors (Ortony, 1987; Elison, 2005) claim that they are two slightly different cognitive specifications of the same basic affective phenomenon, which would explain why some times they are hard to distinguish, and should cast doubts on attempts at sharply differentiating their functions.

Tangney and Dearing's definitions of shame and guilt rely on a clear separation between self and behavior, where “self” refers to the set of features that define an individual. Instead, I believe that selfhood should be conceived as a dynamic process of self-conscious individuation that can rely on different dimensions in different contexts (see, e.g., Zahavi, 2005; Reddy, 2008; Rochat, 2009). According to Tangney and Dearing, in shame, self-individuation takes place in terms of a negative feature that is perceived as defining the self as a whole: for example, greed. I perceive myself as greedy and I am ashamed of myself as a result. In my view, this account overlooks several dimensions of the shame experience that play a crucial role in the process of self-individuation, namely embodiment, situatedness and temporality (Guenther, 2011; Zahavi, 2012; León, 2013): I apprehend myself not simply as *a* (any) greedy individual, but as *this* singular one, *me*, put on the spot here and now. As León (2013, p. 211) puts it, to feel shame is “to experience in intersubjective contexts the irreducibility of one's own particular subjective situation in the world.” Admittedly, these phenomenological dimensions don't render themselves easily to operationalization and testing. But my worry is not so much that descriptions of shame and guilt are inaccurate, but that strong moral conclusions are drawn from them. Behavior often contributes crucially to dynamic and situational self-individuation, so the boundary between them is blurry and permeable. Let me be clear here: I agree that self and behavior are concepts that mark a helpful distinction. But Tangney and Dearing further tell us that, in the interest of morality, we ought to *disconnect* them, that our emotions of shame and guilt do just that, and that a focus on behavior is morally preferable to a focus on self—indeed, it is not merely preferable, it is the morally good choice versus the morally bad choice (see Tangney and Dearing, 2004, esp. ch. 5 and 6). This entails that there are no situations where shame might be the more appropriate moral response, which is questionable (ought citizens of Western countries feel guilty, as opposed to ashamed, of our governments' failure to prevent the genocides in Rwanda and Bosnia, for example? See Hutchinson (2008) and Morgan (2008) on this issue).

A more serious concern is that Tangney and Dearing's very definitions of shame and guilt already imply many of the factors they are trying to test. In particular, the antisocial and destructive nature of shame and the prosocial and constructive nature of guilt are presupposed by and built into their TOSCA tests (see Ferguson and Stegge, 1998; Luyten et al., 2002; Giner-Sorolla et al., 2011; Nelissen et al., 2013, p. 358). Luyten et al. (2002) have shown that the original TOSCA overwhelmingly represents cases of mild, adaptive guilt related to reparation, and maladaptive aspects of shame related to low self-esteem. Drawing on these findings, Giner-Sorolla et al. (2011, p. 446) reach the conclusion that “TOSCA guilt measures the motivation to respond to one's own misdeeds with compensatory action, whereas TOSCA shame measures the tendency to experience intense emotions of guilt and shame from the appraisal of self-blame, and to a lesser extent the desire to withdraw from others.” Thus, the test does not track shame and guilt, but two different ways of dealing with them.

This takes me to another worry: the TOSCA test is designed to measure a disposition or a character trait, *proneness* to feel shame or guilt in various situations, but in the subsequent interpretation of results, Tangney and Dearing extend their conclusions to individual episodes of these emotions. This is problematic, because, as Nelissen et al. (2013, p. 359) explain, the characteristics of the people who are generally predisposed to feel a particular emotion in a wide array of circumstances tell us very little about the *function* and effects of isolated episodes of that emotion in just any person. From the finding that shame-*proneness* is *associated* with low self-esteem one cannot conclude that all individual episodes of shame *lead to* low self-esteem. The conclusion of Tangney and Dearing's study should be that people with certain character traits or dispositions tend to deal with emotions of self-assessment in counterproductive ways, not that shame is destructive and guilt is constructive across the board.

## Insufficient account of the role of others

Further, some important elements to determine whether shame will have productive results or not are contextual and depend on interaction. Indeed, De Hooge et al. (2010) have found in their empirical studies that shame *can*, and actually *does*, lead to prosocial behavior in certain circumstances, namely in dyadic interactions where the partners have witnessed the shameful behavior. If somebody does something shameful in front of us, and we see this person react with shame, our opinion of the offender is likely to be much less negative that if this person acts shamelessly. This is so because, from a second-person perspective, shame reveals a concern for other people's opinions, as well as for shared norms and standards, which can counter the effects of a previous failing and partially restore other people's trust in the offending individual.

Tangney and Dearing disregard this. They combine their functionalistic understanding of morality (behavior is considered moral when it tends to favor others at the expense of oneself) with an agent-centered take on it, which overlooks interaction and group dynamics. Actions are judged as morally constructive if, from the agent's perspective, they are in any measure altruistic or other-regarding, and they are judged as morally counterproductive if the opposite is the case. But no attention is paid to other people's perceptions of and reactions to displays of these emotions, or to the intersubjective interactions that ensue, which can and often do have prosocial consequences. Those tendencies should be part of a functionalistic story about the role of these emotions in morality, but this is not enough. In my view, this type of functionalistic and consequentialist approach is too narrow to fully account for the private aspects of morality (self-evaluation, self-transformation, deliberation and decision-making) and overly simplifies the public ones, reducing them to action tendencies.

Moreover, the abovementioned studies of dyadic interactions only show a small fraction of the important role of others in shame. Rochat (2009) and Seidler (1996, 2000), among others, offer accounts of shame as crucial to the intersubjective development and sustainment of self-consciousness. Shame would precisely be crucial because it captures the experience of self in relation to others and is the product of a discrepancy between the first- and the third-person perspectives on oneself (Rochat, 2009, p. 105, 108, 109). This role in self-constitution is also essential to morality in ways that Tangney and Dearing's account cannot do justice to. It is crucial for self-examination, learning and self-transformation. In my view, the intersubjectivity and social self-consciousness that shame entails constitute a ground from which morality can take off. A *capacity* to feel shame would therefore be morally productive in general, not only in the contingent occasions in which shame actually works to foster harmonious social relations. One of the standard, albeit controversial (see Deonna et al., 2011), claims about shame is that it is a social emotion. In my view, the correct way to interpret this claim is not that in every instance of shame I evaluate myself exactly as the other does—an interpretation that has its own share of problems—, but rather that this emotion entails a widening of my perspective where I recognize that a part of who I am escapes my control and depends on the other (see Sartre, 2003). Shame does not include all the elements that moral goodness requires, but it does attest to our openness to others, our “irreducible relationality” (Guenther, 2012, p. 71), and it can show that we take seriously the shared practice of morality (Calhoun, 2004, pp. 139–146). Before dismissing shame as morally counterproductive, its crucial role in intersubjective self-constitution needs to be studied in its full complexity (see, e.g., Schneider, 1977; Hutchinson, 2008; Reddy, 2008; Williams, 2008; Rochat, 2009; Guenther, 2011; Zahavi, 2012; León, 2013; Welz, 2014). TOSCA-based research programs overlook or flatten many of these issues, and therefore can only offer a limited picture of the role of shame and guilt in morality.

### Conflict of interest statement

The author declares that the research was conducted in the absence of any commercial or financial relationships that could be construed as a potential conflict of interest.
